# Does intervention engagement mediate physical activity change in a web-based computer-tailored physical activity intervention?—Secondary outcomes from a randomised controlled trial

**DOI:** 10.3389/fdgth.2024.1356067

**Published:** 2024-05-21

**Authors:** Corneel Vandelanotte, Camille E. Short, Ronald C. Plotnikoff, Stephanie Schoeppe, Stephanie J. Alley, Quyen To, Amanda L. Rebar, Mitch J. Duncan

**Affiliations:** ^1^ Physical Activity Research Group, Appleton Institute, Central Queensland University, Rockhampton, QLD, Australia; ^2^ Melbourne Centre for Behaviour Change, Melbourne School of Psychological Science and Melbourne School of Health Science, University of Melbourne, Melbourne, VIC, Australia; ^3^ Centre of Active Living and Learning, College of Human and Social Futures, University of Newcastle, Newcastle, NSW, Australia; ^4^ School of Medicine and Public Health, College of Health, Medicine and Wellbeing, The University of Newcastle, Newcastle, NSW, Australia; ^5^ Active Living Research Program, Hunter Medical Research Institute, Newcastle, NSW, Australia

**Keywords:** eHealth, exercise, intervention usage, online, internet, video, tailored

## Abstract

**Introduction:**

The relationship between intervention engagement and behaviour change may vary depending on the specific engagement metric being examined. To counter this composite engagement measures may provide a deeper understanding of the relationship between engagement and behaviour change, though few studies have applied such multidimensional engagement metrics. The aim of this secondary analysis of RCT data was to examine how a composite engagement score mediates the effect of a web-based computer-tailored physical activity intervention.

**Methods:**

501 inactive Australian adults were randomised to a no-treatment control or intervention group. Intervention participants received 8 sessions of web-based personalised physical activity advice over a 12-week intervention period and the ability to complete action plans. Change in physical activity was assessed using Actigraph accelerometers at baseline, 3-months and 9-months. Engagement with the intervention (i.e., a composite score including frequency, intensity, duration and type) was continuously assessed during the intervention period using website tracking software and database metrics. Generalised structural equation models were used to examine how a composite engagement score mediated intervention effects at 3 months and 9 months.

**Results:**

At 3 months, mediation analysis revealed that the intervention group had significantly higher engagement scores than the control group [a-path exp(b) = 6.462, 95% CI = 5.121–7.804, *p* < 0.001]. Further, increased engagement with the intervention platform was associated with an increased time spent in moderate-to-vigorous physical activity [ab-coefficient exp(b) = 1.008, 95% CI = 1.004–1.014, *P* < 0.001]; however, the magnitude of this effect was small. There were no significant mediation effects at the 9-month time point.

**Discussion:**

The findings suggest that a composite intervention engagement score has a small positive influence on physical activity changes and that other factors (e.g., behaviour change techniques) are likely to be more important drivers of behaviour change.

## 1 Introduction

Regular physical activity lowers the risk of developing non-communicable diseases, such as cardiovascular disease, some types of cancer and diabetes (1, 2). Physical activity has also shown to improve mental health outcomes and prevent weight gain (3). However, the population prevalence of physical inactivity in Australia and around the world is high, with up to half of the population not meeting recommended minimum guidelines to improve health outcomes (4, 5). As such, the search for affordable, scalable, and effective physical activity interventions is ongoing (6). As broadband Internet access is high in many countries (e.g., 90% in Australia), using the Internet for delivering web-based physical activity interventions has the potential to be cost-effective and wide-reaching (7).

While systematic reviews and meta-analyses have demonstrated positive outcomes for web-based physical activity interventions (8, 9), the effects on physical activity are most often small and short-lived, which is partially driven by low user engagement with these interventions (10, 11). However, web-based interventions that provide ‘tailored’ or individually adapted physical activity advice (i.e., computer-tailored interventions) have demonstrated improved effectiveness compared with interventions offering generic or targeted information (12). Computer-tailored interventions are underpinned by the Elaboration Likelihood Model which poses that providing personalised information leads users to pay more attention to intervention content and deeper processing of this information, which in turn leads to the intervention being more effective (13). As such, one would also expect greater user engagement in web-based computer-tailored interventions, as compared to generic or targeted web-based interventions (14). Unfortunately, engagement with web-based computer-tailored interventions is rarely reported, and thus relatively little is known about how participants use and engage with these types of interventions.

Understanding the way in which participants use and interact with web-based interventions is important to improve their design and effectiveness. In the behavioural science literature, engagement has typically been conceptualised as “usage” of digital behaviour change interventions, focusing on the temporal patterns (e.g., frequency, duration) and depth of usage (e.g., use of specific intervention content) of usage (15). Although there is a prevailing assumption that increased engagement is associated with a greater likelihood of behaviour change, a recent systematic review has shown that the strength of this relationship appears to be weak (14, 16). Furthermore, it is commonly observed that engagement tends to decline over the course of the intervention (6, 17). Additionally, discrepancies exist between studies concerning the conceptualization and measurement of engagement, which makes comparisons between studies difficult (15, 18–21). A systematic review has indicated that a more favourable subjective user experience, completion of a greater number of activities, and more frequent logins consistently relate to increased physical activity; while time spent on the website is not necessarily linked to physical activity (17).

The relationship between engagement with the intervention and behaviour change may vary depending on the specific engagement metric being examined and that single engagement metrics may not comprehensively capture how participants use and engage with the intervention. In addressing this, Short and colleagues (19) have proposed that frequency (i.e., the number of self-monitoring entries or logins), intensity (i.e., the number of intervention features utilized), duration, and type (i.e., reflective, didactic, or active) of usage should all be considered when examining engagement. This can be done by using a composite measure that encompasses all these engagement components (22). Such composite measures may offer a more valuable perspective for understanding the relationship between engagement and behaviour change (18–20), although relatively few studies have implemented such multidimensional measures.

Therefore, the aims of this secondary analysis of data from a randomised controlled trial were to examine the effect of a web-based computer-tailored intervention on a composite measure of intervention engagement relative to a control group, and to examine how the composite engagement measure mediates the effect of the intervention on physical activity.

## 2 Materials and methods

### 2.1 Study design

The TaylorActive intervention was a 3-group randomized controlled trial conducted at Central Queensland University in Rockhampton, Australia. The trial protocol and main outcomes have been described in depth elsewhere (23, 24). Participants were randomized into 3 groups: video-tailored intervention, text-tailored intervention, and control. Trial assessments were conducted at baseline, 3 months, and 9 months. However, the user engagement data presented in this study were all collected between the baseline and the 3-month assessment point, as this is when the active intervention phase of the study took place. All groups received access to a website with a text-based library with generic physical activity information. The control group had no access to other website components and was regarded as ‘usual care’ condition. The text-tailored and video-tailored groups also gained access to eight personally tailored physical activity sessions (delivered over 12 weeks) and an action planning tool. The sole difference between both intervention groups was how the tailored information was delivered: as tailored text on a webpage in the text-tailored group, or as tailored online videos in the video-tailored group. The trial did not find significant physical activity differences between intervention groups (24), nor were any significant differences observed in terms of engagement with the website. As such, the intervention groups were pooled into one group for the purpose of this secondary analysis. Therefore, this study will only report on two groups: control and intervention (i.e., combined text/video groups). All participants provided informed consent and ethical approval for the trial was granted by the Human Research Ethics Committee of the Central Queensland University (reference number: H14/07–163).

### 2.2 Participants

Eligible participants were aged 18+ years, had broadband Internet access, could speak and read English, were living in Australia, were insufficiently physically active (i.e., 150 min of moderate-to-vigorous physical activity per week) (25), self-reported that it was safe for them to increase physical activity by answering “no” to all questions on the Physical Activity Readiness Questionnaire (26) or obtained medical clearance, were not pregnant, had a body mass index over 17.5, and reported no impairments that could prevent them from becoming more active. Participants were recruited through social media advertisements (i.e., Facebook), traditional media (e.g., radio, The Conversation), email (e.g., Central Queensland University staff), and third-party databases (i.e., www.trialfacts.com). Interested individuals were directed to a recruitment webpage that contained detailed study information and asked them to complete a screening survey to determine their study eligibility.

Project officers contacted potential participants after completing the screening tool via telephone to verify eligibility and contact details and then posted them an accelerometer with and a return postbag. Participants were asked to wear the accelerometer for seven consecutive days. Randomisation, using a randomly generated sequence via www.randomization.com, occurred once baseline data were obtained. Accelerometer procedures were repeated at 3-month and 9-month assessments. There was no face-to-face contact with participants at any time during the study.

### 2.3 Intervention

An in-depth description of the intervention is available elsewhere (23). Briefly, the intervention aimed to increase all domains of physical activity (e.g., leisure, active travel, house/garden work, occupation). A library, available to all groups, contained 19 articles about different aspects of physical activity (e.g., “Why be active”, “Get started walking”, “Make time to be active”). The eight sessions of tailored physical activity content, available to both the text-tailored and video-tailored groups, was generated in response to brief online questionnaires about physical activity in conjunction with questions relating to evidence-based individual, social and environmental determinants of physical activity. IF-THEN algorithms were applied to select personally relevant advice from a comprehensive database. Health behaviour theories used to inform intervention content included self-determination theory (27), social cognitive theory (28) and theory of planned behaviour (29). The following constructs were addressed throughout the sessions: self-efficacy, intentions, social support, knowledge, outcome expectancies, attitudes, facilitators and barriers and risk perception, intrinsic and extrinsic motivation, need for relatedness, peripheral and central cues and habits. The following behavioural change techniques were applied to change theoretical constructs: feedback, self-monitoring, goal setting, habit formation, instruction, problem solving and action planning. Physical activity advice and goals were tailored to participants’ main motivation to increase activity levels: (1) improve health, (2) increase fitness, (3) increase strength, (4) lose weight and (5) reduce stress. The eight sessions with tailored feedback were delivered in a set order at a set time. New sessions could only be accessed when previous sessions had been completed. The first four intervention sessions were delivered every 7 days (month one); the next four sessions were delivered every 14 days (months two and three). As such, the total length of the program was 12 weeks (three months).

A website feature for creating physical activity action plans was also available for both intervention groups (30). At the end of each session (except for the first and last) participants were asked to set short-term physical activity goals and to create an action plan for how they would meet those goals (30). For example, participants were asked very specific questions about how they planned to meet their physical activity goals: What physical activity they will do, where they will do it, when they will do it, how often they will do it, how long will each activity session be, and with whom they will do it (31). When participants had completed all the questions, the TaylorActive website provided an action plan that could be printed on a single page.

### 2.4 Measures

#### 2.4.1 Physical activity

At each assessment time point, moderate-to-vigorous physical activity (min/week) was assessed by hip-worn ActiGraph GT3X + activity monitors during all waking hours over 7 days (32). Monitors were configured to collect triaxial acceleration data at a sampling frequency of 30 Hertz, but downloaded as 1 s epochs and aggregated to 60 s epochs using Actilife software (V.6.13.3). Valid wear time was defined as ≥10 h on ≥5 days within a 7-day period (33). Non-wear time was assessed using the Choi et al. algorithm (vector magnitude) and was defined as 90 consecutive minutes of 0 counts per minute, allowing for a 2 min interruption (34). moderate-to-vigorous physical activity was defined as ≥2,690 counts per minute (vector magnitude) (32).

#### 2.4.2 Intervention engagement

A composite measure of intervention engagement was developed to capture intervention group participants’ frequency, intensity, duration, and type of engagement with the intervention platform (22). Data for this score was derived from two sources: (1) the intervention website database (e.g., number of sessions completed) and (2) Google Analytics website traffic platform (e.g., time spent on a specific page of the website). This objective data was collected continuously from the moment participants first accessed the website until they completed the intervention period after 12 weeks (3-months). All data for the composite engagement measure were aggregated on a week-by-week basis.

The four dimensions of the composite engagement measure were assessed as follows:

1. Frequency: the number of times participants accessed the website per week (i.e., number of visits)
2. Intensity: a four-item score composed of the following sub-dimensions:
  a. Time (sec/week) reading (or viewing) the personalised feedback from the 8 activity sessions
  b. Time (sec/week) reading the 6 action plans
  c. Completion status of the session on a scheduled week
  d. Completion status of the action plan on a scheduled week
3. Duration: the total time spent on the website each week
4. Type: the number of library articles read each week

For each week, the frequency (1), the 4 sub-dimensions of the intensity component (2a–d), duration (3) and type (4) received a score from 0 to 10, where a higher score indicated a higher level of engagement. However, due to the different metrics used for each engagement dimension, all with different ranges, we had to rescale all scores to be between 0 and 10. This formula was used to do this: $\mathrm{rescaled}\;\mathrm{score}\;=\left(\frac{X-X_{\min}}{X_{\mathrm{range}}\;}\right)n$; where *X* is the original score, *X*_min_ is the minimum of the observed variable, *X*_range_ is the range of the potential score and *n* is the upper limit of the rescaled score [10]. Prior to each dimension being rescaled, values larger than the mean value plus two standard deviations were truncated to limit the impact of potential outliers. Next, the intensity sub-dimensions (scales 2a–d) were averaged to a single scale also ranging from 0 to 10. As such, a weekly engagement score was created as the sum of the four dimensions with a range of 0–40 and the total composite engagement score over 12 weeks was the sum of weekly engagement scores ranging from 0 to 480.

#### 2.4.3 Demographics

Sociodemographic characteristics included in this study were gender, age, years of schooling, self-reported Body Mass Index (BMI), relationship status, living environment, employment status and income. Participants’ weekly household income was categorised as: (1) less than AUD $1,250 per week; (2) between AUD $1,250 and 2,000 per week; (3) between AUD $2,000 and 3,000 per week; (4) more than AUD $3,000 per week. BMI was calculated as weight in kilograms divided by height in meters squared and interpreted based on the standard weight status categories: Underweight (<18.5 kg/m^2^), Health Weight (18.5–<25 kg/m^2^), Overweight (25.0–<30 kg/m^2^) and Obese (≥30.0 kg/m^2^). Accelerometer assessed MVPA was dichotomised (</≥150 min/week) to reflect meeting the National Physical Activity Guidelines (35).

### 2.5 Statistical analysis

The analysis followed the intention-to-treat (ITT) principle, specifying that data from all those who were randomised were analysed. To ensure that the ITT population was analysed, missing baseline data on the ActiGraph measures (*n* = 38) were mean imputed. Differences between the three original study groups on the composite engagement score were examined using a Kruskal–Wallis test with follow-up comparisons between the groups using Wilcoxon rank-sum tests.

**Figure 1:**
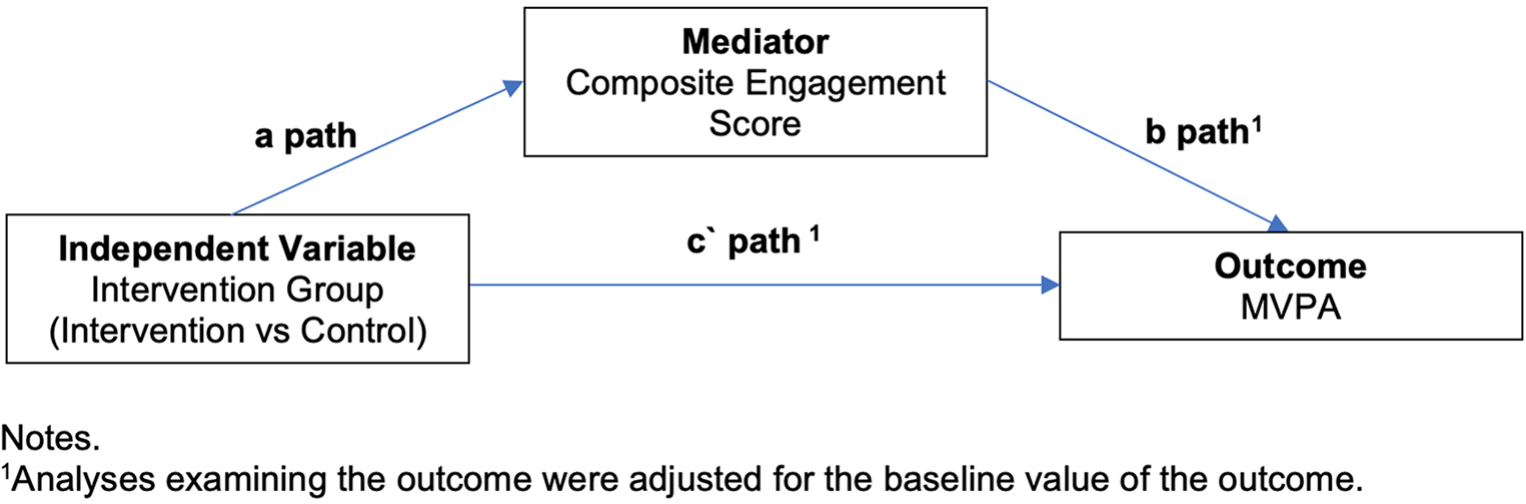
Overview of the mediation model.

Generalised structural equation models were used to examine intervention effects on the composite engagement score, the intervention effects on physical activity, and how the composite engagement score mediated intervention effects at 3 months and 9 months. Figure 1 outlines the mediation model used. It is common to adjust analyses of physical activity from accelerometer data for wear time, however in the current models the proportion of wear time spent in moderate-to-vigorous physical activity was used as the outcome to account for differences in wear time between individuals and overcome the need to include wear time as a covariate in the structural equation models. As the mediator, the composite engagement score was only assessed during the intervention period, therefore it was not possible to adjust the mediation model for the baseline value of the mediator as is commonly performed (36–42) The a-path represents the effect of the intervention on the hypothesised mediator, the b-path represents the association between the mediator and the outcome variables, and the c`-path represents the direct effect of the intervention on the outcome after adjusting for the mediator. Mediation effects were estimated using a product-of-coefficients approach (denoted by a*b) and the 95% confidence intervals of the ab-coefficient were estimated using bias-corrected bootstrapping on 5,000 samples (36, 37). The c`-path represents the total effect of the intervention on the outcome (i.e., ab + c`). The b-path and c`-paths were adjusted for the baseline value of the outcome. Missing data at follow up was assumed to be missing at random and maximum likelihood was used to handle missing data and include all available observations.

To inform the choice of model and link, residual diagnostics were used in a series of separate generalised linear models for each path. Consistent with previously published analyses of this trial (24), a gamma model with log link was selected. Results are presented as exponentiated coefficients and coefficients (except for the ab-coefficient) are interpreted as the percentage change in the outcome given a one unit in the independent variable. The assumption of no exposure (i.e., study group) and interaction between group and mediator outcome was examined using generalised linear models and no significant interaction was observed at 3-months (*p* = 0.805) or 9-months (*p* = 0.0861). Analyses were conducted using Stata MP 17 and applying an alpha level of 0.05.

## 3 Results

Table 1 shows participant baseline data. The majority of participants (*n* = 501) were female (72%), with an average age of 44 (±13) years and had 14 or more years of schooling (78%). More than 80% of participants were employed, over 60% lived in a major city and over 65% were overweight or obese.

**Table 1:** Sample characteristics at baseline.

	Control (*n* = 167)		Intervention (*n* = 334)	
	*n*	%	*n*	%
Gender				
Male	46	28	94	28
Female	121	72	240	72
Age (year)				
18–<45	85	51	175	52
45–<65	69	41	141	42
≥65	13	8	18	5
Years of schooling				
0–<14	33	20	76	22
14–<21	117	70	216	65
≥21	17	10	42	13
BMI (kg/m^2^)				
Underweight (<18.5)	1	1	4	1
Healthy weight (18.5–<25.0)	41	25	118	36
Overweight (25.0–<30.0)	55	33	99	30
Obese (≥30.0)	69	42	111	33
Relationship status				
Single	29	17	75	22
Widowed/divorced/separated	19	11	31	9
Married/de facto	119	71	228	68
Living environment				
Major city	110	66	196	59
Regional/remote	57	34	138	41
Employment status				
Full time	88	53	173	52
Part time/casual	51	31	108	32
Unemployed/retired/others	28	17	52	16
Income (AUD)				
≥3,000/week	40	26	61	22
2,000–<3,000/week	42	27	76	28
1,250–<2,000/week	38	25	83	31
<1,250/week	33	22	52	19
Meeting physical activity guidelines (150 min/week) MVPA				
Not meeting	136	81	258	77
Meeting	32	19	77	23

To provide context in terms of how the intervention was used, Table 2 presents participant engagement metrics by group. Participants in the pooled intervention group visited the website 7.5 ± 6.4 times during the 12-week intervention period, compared to 1.4 ± 1.9 times in the control group. Intervention participants spent 83.5 ± 76.1, minutes in total on any part of the website and of this time they spent 23.6 ± 24.0 min on the personalised advice and they spent 2.8 ± 5.5 min the action plan. The control participants spent a total 8.5 ± 16.1 min in total on any part of the website (and did not have access to the other parts of the website). On average, intervention participants completed nearly 5 intervention sessions, nearly 3 action plans and read nearly 2 articles from the website library.

**Table 2:** Engagement metrics by group during the 12-week intervention period.

	Pooled intervention groups (*n* = 334)	Text-tailored intervention group (*n* = 167)	Video-tailored intervention group (*n* = 167)	Control group (*n* = 167)
Frequency: total number of website visits	7.5 ± 6.4	7.3 ± 5.4	7.6 ± 7.2	1.4 ± 1.9
Intensity 1: total time (minutes) reading/viewing personalised advice	23.6 ± 24.0	15.9 ± 17.65	31.4 ± 27.0	0.0 ± 0.0
Intensity 2: total time (minutes) reading Action Plan	2.8 ± 5.5	2.6 ± 5.8	3.0 ± 5.3	0.0 ± 0.0
Intensity 3: Total number of sessions completed (maximum = 8)	4.9 ± 2.8	5.0 ± 2.8	4.8 ± 2.8	0.0 ± 0.0
Intensity 4: Total number of Action Plans completed (maximum = 6)	2.7 ± 2.1	2.7 ± 2.2	2.7 ± 2.1	0.0 ± 0.0
Duration: total time (minutes) spent on the website	83.5 ± 76.1	77.0 ± 80.0	90.1 ± 71.8	8.5 ± 16.1
Type: total number of library articles read (maximum = 19)	1.8 ± 3.7	1.8 ± 4.0	1.7 ± 3.4	4.1 ± 6.3

**Figure 2:**
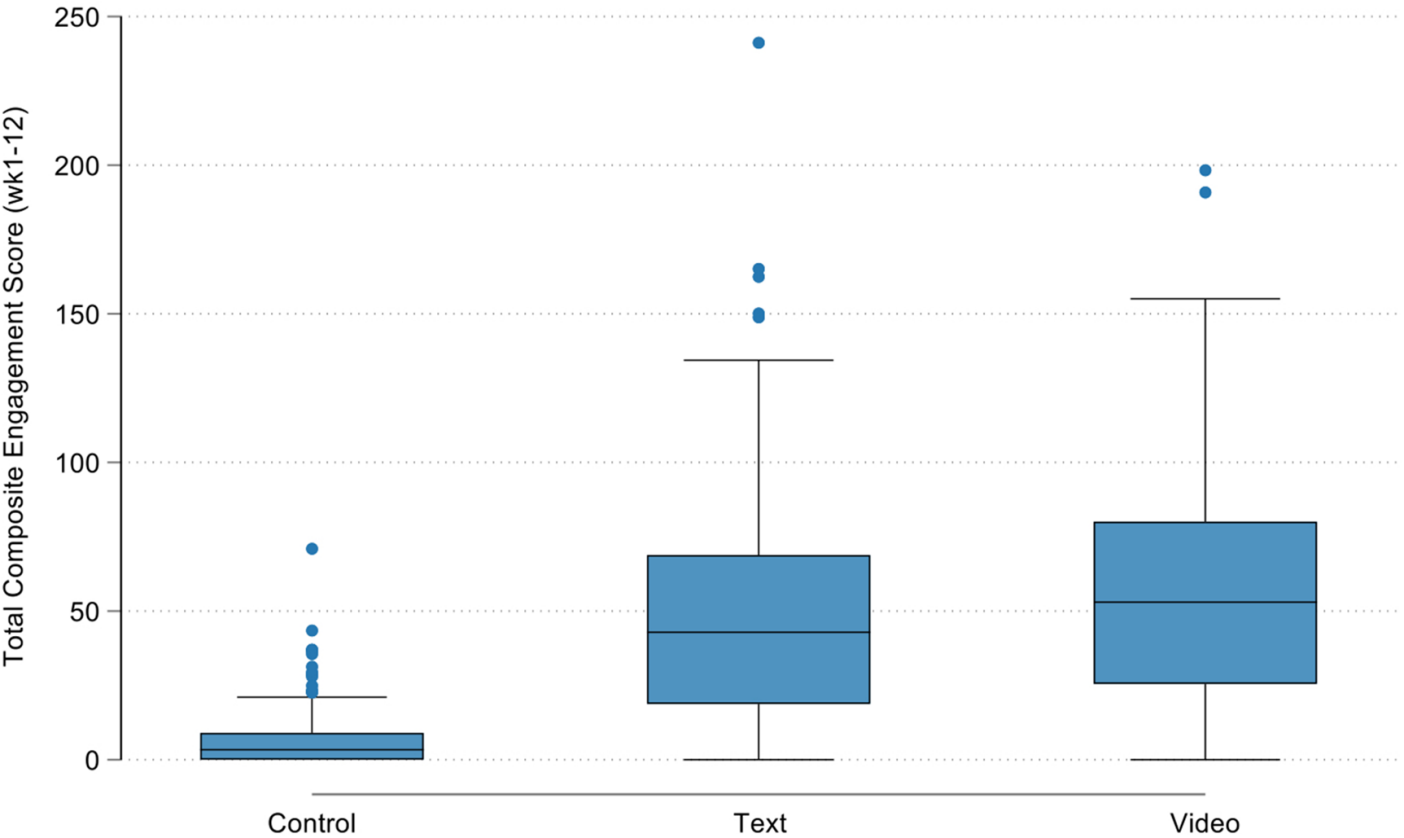
Total composite engagement score prior to pooling text- and video-tailored intervention groups.

**Figure 3:**
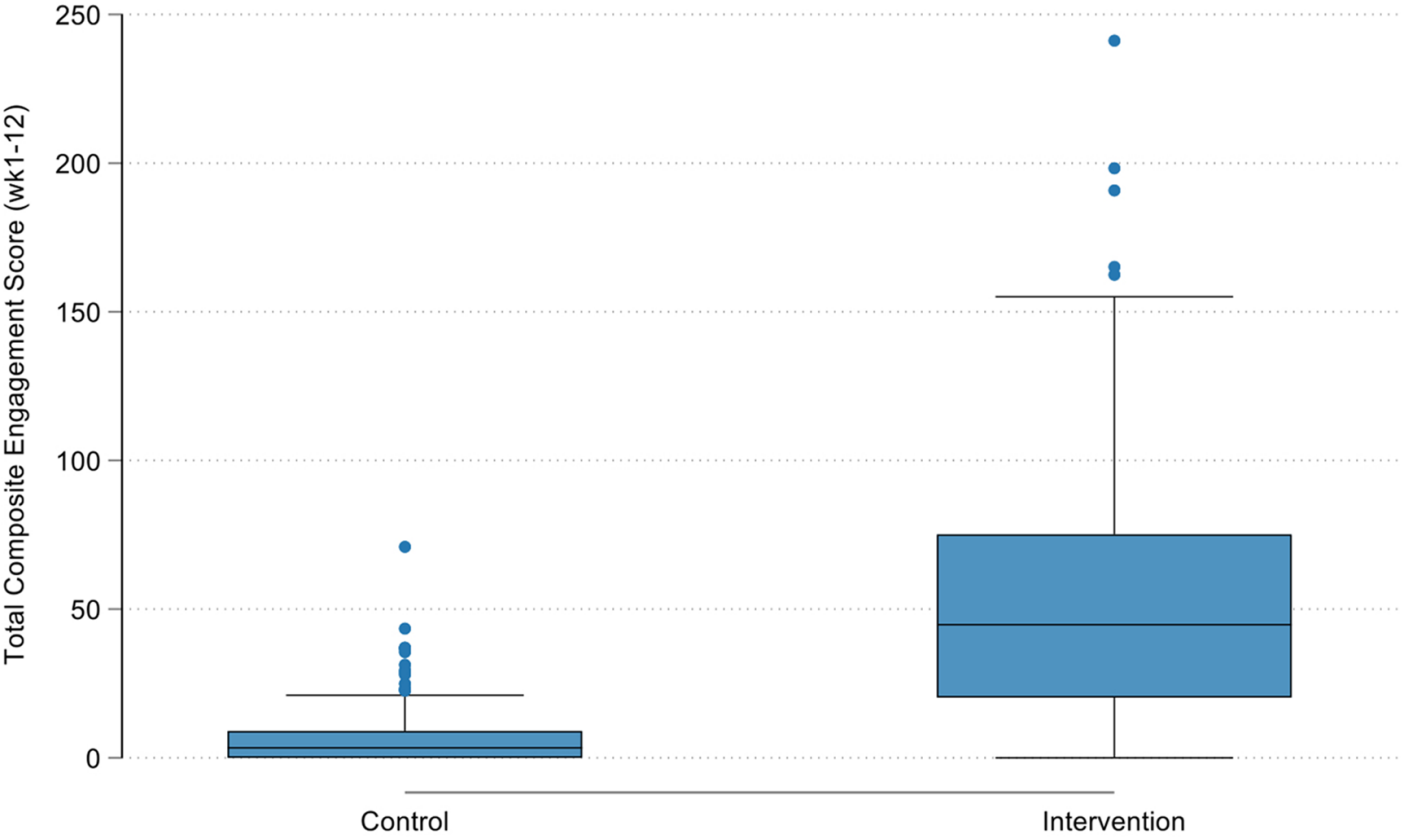
Total composite engagement score after pooling text- and video-tailored intervention groups into a single group.

**Figure 4:**
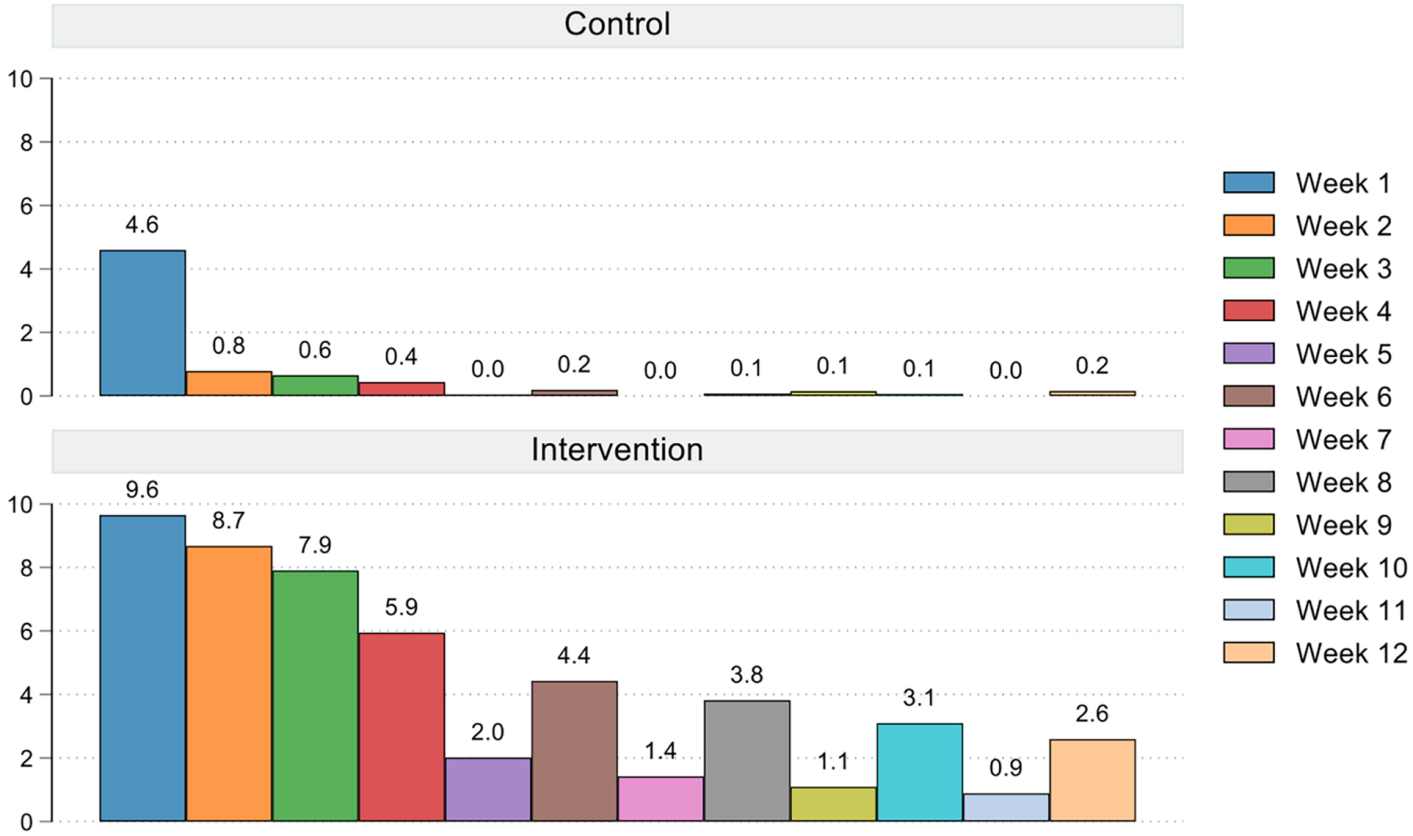
Average weekly composite engagement score for the pooled intervention group and control group.

Figure 2 shows the total composite engagement score by the three original study groups (i.e., video-tailored intervention, text-tailored intervention, and control group) highlighting the similarities in engagement score between the text- and video-tailored groups. These differences are supported by the results of the Kruskal–Wallis test indicating that overall there was a difference between groups in engagement scores (*p* = 0.001), and Wilcoxon rank-sum tests indicating that these differences were between the control and text-tailored group (*p* < 0.001), control and video-tailored group (*p* < 0.001) and not between text- and video-tailored groups (*p* = 0.9205). Figure 3 shows the total composite engagement score for the pooled intervention group (text- and video-tailored combined) and the control group. Figure 4 shows the weekly engagement scores for the pooled intervention group and the control group.

At 3 months, mediation analysis revealed that there were significant direct {c` [exp(b) = 0.708, se = 0.094, 95% CI = 0.523–0.893, *p* = 0.010]} and total effects {c [exp(b) = 0.714, se = 0.094, 95% CI = 0.530–0.898, *p* = 0.011]} of the intervention on the proportion of time spent in moderate-to-vigorous physical activity, indicating that at 3-months the intervention groups spent a smaller proportion of time in moderate-to-vigorous physical activity than the control group. The intervention group had significantly higher engagement scores, indicating greater engagement than the control group {a-path [exp(b) = 6.462, se = 0.684, 95% CI = 5.121–7.804, *p* < 0.001]}, and greater engagement scores were associated with greater proportion time in moderate-to-vigorous physical activity {b-path [exp(b) = 1.005, se = 0.001, 95% CI = 1.002–1.007, *p* = 0.001]}. Mediation results suggest that greater engagement with the intervention platform during the 12-week intervention period was associated with an increased proportion of time spent in moderate-to-vigorous physical activity at the 3-month assessment time point {ab [exp(b) = 1.008, se = 0.003, 95% CI = 1.004–1.014, *p* < 0.001]}, however increased engagement only led to very small increases in physical activity.

At 9 months, mediation analysis revealed that there were no significant direct {c` [exp(b) = 1.047, se = 0.184, 95% CI = 0.687–1.41, *p* = 0.793]} and total effects {c [exp(b) = 1.05, se = 0.181, 95% CI = 0.690–1.400, *p* = 0.798]} of the intervention on the proportion of time spent in moderate-to-vigorous physical activity. The intervention group had significantly higher engagement scores, indicating greater engagement, than the control group {a-path [exp(b) = 6.462, se = 0.684, 95% CI 5.121–7.804, *p* < 0.001]}, and engagement scores were not associated with the proportion of time in moderate-to-vigorous physical activity {b-path [exp(b) = 0.999, se = 0.002, 95% CI 0.995–1.003, *p* = 0.606]}. Mediation results suggest that intervention engagement during the 12-week intervention period did not mediate changes in the time spent in moderate-to-vigorous physical activity at 9 months {ab [exp(b) = 0.998, se =0.004, 95% CI 0.992–1.006]}.

## 4 Discussion

This secondary analysis of data from a randomised controlled trial examined whether engagement with a web-based computer-tailored intervention mediated change in physical activity. Overall, the findings showed that the pooled intervention groups were significantly more engaged with the intervention than the control group, and this higher engagement was associated with increased time spent in moderate-to-vigorous physical activity at 3 months but not at 9 months post-baseline.

Overall, engagement with the website declined strongly within the first couple of weeks in all study groups. While intervention participants continued to engage with the website throughout the 12-week intervention period, this was not the case (as anticipated) in the control group, where engagement almost completely ceased after week one. These findings are comparable to other web-based physical activity interventions that report sharp declines in intervention engagement (43–46), with similar “waterfall” shaped engagement graphs, whereby the majority of participants only engage with the intervention for a couple of weeks and whereby after a sudden drop only a small minority of participants keeps engaging until the end of the intervention period. The relatively modest engagement with the intervention website in this study can partially be explained by its design. The timed release of new intervention sessions (i.e., weekly for the first 4 weeks and biweekly for the next 8 weeks) encouraged infrequent use of the website. In between the release of new sessions participants had few incentives to visit the website, and this ‘up-and-down’ engagement pattern is also visible in the composite engagement score shown in Figure 4. Engagement with the website might have been higher if it offered additional features, such as continuous self-monitoring through activity trackers, social networking, or gamification and rewards; all features that have shown to increase physical activity motivation and engagement (47). The features on the intervention website were purposefully kept minimal to be able to examine the effect of the personalised feedback in isolation. However, a subsequent pilot-study that we conducted using the same website with integrated Fitbit activity trackers, did show much improved engagement in the Fitbit intervention group, with twice as many participants completing all intervention sessions compared to the intervention group without Fitbits (48).

While our study did find that higher engagement with the intervention was associated with more time spent in moderate-to-vigorous physical activity, the results also indicated that the magnitude of this mediation effect was small, and it only occurred at 3-months, not at 9-months. These findings align with a meta-analysis examining the association between engagement with a web-based intervention and change in physical activity (17). This study demonstrated that while there is a statistically significant positive relationship between engagement and physical activity, it was also a weak association [b = 0.08, 95% CI = 0.01–0.14] (17). This suggests that while engagement with a digital intervention is important for achieving behaviour change, it may not be the major driver of behaviour change (18, 22). How engagement impacts on the process of behaviour change (some types of engagement may have more impact than others) is an important consideration. This aligns with conceptual frameworks of engagement and behaviour change indicating that engagement with a digital intervention is only one of several important factors necessary to actually achieve behaviour change (49). Other factors include psycho-social factors related to behaviour change (e.g., motivation, social support), personal relevance of the information provided, and the inclusion of effective behaviour change techniques in the intervention (13, 18, 19). This means that developing digital interventions to maximise engagement only will not be sufficient achieve sustainable behaviour change, they will also need to include features associated with successful behaviour change. As such, the importance of engagement is relative to the effectiveness of the intervention components and behaviour change techniques used. It also means that, as yet, we do not know what the optimal amount of intervention engagement is (50). It is likely to be influenced by a range of factors such as characteristics of participants themselves, the targeted outcome (e.g., physical activity vs. diet) and the design of the intervention itself (e.g., session based vs. continuous monitoring) (50). The latter will determine what the ‘intended’ engagement is with the digital intervention. In this context, it is reasonable to assume that no digital health intervention is intended to be used forever. If the intervention is successful in achieving its goals (i.e., behaviour change), then at some point participants will either reduce or stop engaging with the intervention because they have learned all the skills and knowledge needed to engage in the behaviour without external support (22). As such, a natural decline of user engagement should be expected, however, this decline is likely to be slower and more gradual than what is experienced in most digital interventions to date (i.e., “waterfall” shaped drop in engagement described above) (43–46). This indicates more research is needed in this space and we should also investigate what type of digital interventions require the least amount of engagement while still being effective.

A strength of this study is that it entirely relied on device (i.e., Actigraph to measure physical activity) or software (i.e., Google Analytics, website database) assessed data to analyses the outcomes. Avoiding the use of self-reported data (which is very common in similar physical activity studies) limits the potential for bias and enhances the robustness of the study findings. Additionally, while the original RCT was powered to detect statistically significant difference in the primary outcome (i.e., change in physical activity), and not to examine the relationship between intervention engagement and behaviour change; the study sample (*n* = 501) was large and able to examine associations presented in this study. However, a limitation of this study was that the trial sample was not entirely representative of the target population. For example, the trial targeted adults who did not meet the Australian physical activity guidelines (i.e., 150 min of moderate-to-vigorous physical activity per week). Despite screening participants for inactivity during recruitment, 23% of participants met the guidelines at baseline when measured using accelerometery (24, 51). It is harder to achieve physical activity increases in those who are already active, and this reduced the opportunity for the intervention to achieve change. Similarly, already active participants may not have found the intervention very useful (as not designed for them) and therefore engaged less with it. Additionally, the trial sample included a high proportion of females (72%) and highly educated (80%) participants. Finally, there were imbalances between intervention groups in terms of baseline physical activity levels. This may have influenced outcomes, though was accounted for by adjusting the statistical analyses for the baseline value of the outcome.

In conclusion, compared to the control group, the intervention resulted into a significantly higher composite engagement score, which in turn was associated with a small but significant increase in moderate-to-vigorous physical activity immediately after the 12-week intervention, but not 6-months after the end of the intervention. These findings suggest that a composite intervention engagement score has a small influence on physical activity changes and that other factors (e.g., behaviour change techniques, motivation, social support from friends/families) are likely to be more important drivers of behaviour change.

## Data Availability

The data supporting this article will be made available by the authors upon reasonable request.
